# Recovery of dynamic stability during slips unaffected by arm swing in people with Parkinson’s Disease

**DOI:** 10.1371/journal.pone.0249303

**Published:** 2021-04-06

**Authors:** Tarique Siragy, Allen Hill, Julie Nantel

**Affiliations:** School of Human Kinetics, University of Ottawa, Ottawa, ON, Canada; São Paulo State University (UNESP), BRAZIL

## Abstract

The arm elevation strategy assists in recovering stability during slips in healthy young and elderly individuals. However, in people with Parkinson’s Disease, one of the main motor symptoms affecting the upper limbs is reduced arm swing which intensifies throughout the course of the disease before becoming absent. This holds direct implications for these individuals when encountering slips as the arm elevation strategy is an integral component in the interlimb slip response to restore stability. Arm swing’s effect in recovering from slips in people with Parkinson’s Disease though remains unexamined. Twenty people with Parkinson’s Disease (63.78 ± 8.97 years) walked with restricted and unrestricted arm swing conditions on a dual-belt treadmill where slips were induced on the least and most affected sides. Data were collected on the CAREN Extended System (Motek Medical, Amsterdam, NL). The Margin of Stability, linear and angular trunk velocities, as well as step length, time, and width were calculated. Data were examined during the slipped step and recovery step. The restricted arm swing condition, compared to unrestricted, caused a faster step time during the slipped step. Compared to the most affected leg, the least affected had a wider step width during the slipped step. During the recovery step, the least affected leg had a larger anteroposterior Margin of Stability and longer step time than the most affected. No differences between our arm swing conditions suggests that the normal arm swing in our participants was not more effective at restoring stability after an induced slip compared to when their arm motion was restricted. This may be due to the arm elevation strategy being ineffective in counteracting the slip’s backward destabilization in these individuals. Differences between the legs revealed that our participants were asymmetrically impaired in their slip recovery response.

## 1 Introduction

One of the primary concerns for people with Parkinson’s Disease is the increased risk of falling during walking [1–5]. Research from static postural perturbations demonstrate that people with Parkinson’s Disease are particularly susceptible to backward stability loss, which may increase their risk of falling when encountering slips [6]. This is concerning as slips are one of the primary contributors to falling in individuals over the age of 65 [7–9]. However, while gait impairments that contribute to falls in people with Parkinson’s Disease are well documented during unperturbed conditions, evidence during perturbed walking conditions is sparse [10–15].

When first encountering an unexpected slip, healthy young and elderly adults rely on rapid reactive recovery responses to mitigate the destabilizing effects of the perturbation [16–21]. For instance, when the perturbed leg contacts the slipping surface, the knee and ankle joints flex to lower the Center of Mass (COM) to the ground [17, 18]. However, while this flexing response is a strategy to increase stability, extension occurs in the ipsilateral hip due to the hamstring contraction which flexes the knee [18]. This subsequently causes trunk extension and causes the COM to further fall posteriorly (backward) [18]. Thus, to dissipate the COM’s posterior motion, the arms flex forward and upward in an arm elevation strategy to move the COM in the opposing anterior direction [16–18]. Meanwhile, in the unperturbed (recovery) limb, the hip and knee extend to bring the leg that is entering the swing phase rapidly to the ground to increase the base of support (BOS) [16]. After slip exposure, individuals walk with a “cautious gait” strategy where step time and width are increased, and step length is reduced [20, 22, 23]. This is accompanied by a shift in the dynamical state (position and velocity) of their COM anteriorly and away from the mediolateral edges of their base of support at heel-strike [10, 18, 20].

However, in people with Parkinson’s Disease, the multi-system neurodegeneration that occurs, impedes the ability to execute appropriate responses to sudden environmental changes [6]. For instance, evidence from static postural perturbations demonstrates that people with Parkinson’s Disease execute responses that are slower, reduced in amplitude, and have an incorrect directionality with respect to where the perturbation is acting [6]. Additionally, one of the characteristic aspects of idiopathic Parkinson’s Disease is the asymmetric neurodegeneration that affects mobility in one limb (most affected) to a greater extent than the other (least affected) [24, 25]. Evidence from steady-state walking proposed that the least affected leg is more capable of adjusting foot placement at ground contact than the most affected [15]. Thus, the ability to execute recovery responses to slips, may equally be asymmetrically impaired in people with Parkinson’s Disease. The role of both of these limbs in response to slips in people with Parkinson’s Disease, however, has not been determined.

Additionally, the role of arm swing in response to slips in this demographic is currently lacking. In people with Parkinson’s Disease, one of the main motor symptoms affecting the upper limbs is reduced arm swing which intensifies throughout the course of the disease before becoming completely and bilaterally absent [26, 27]. This holds direct implications for these individuals when encountering slips as the arm elevation strategy is an integral component in the interlimb slip response to restore stability [16–19]. However, previous research on restricted arm swing on healthy adults suggests that when the arms are restricted, the upper extremity’s mass is concentrated around the trunk [28]. This subsequently is suggested to increase trunk inertia which in turn increases resistance to changes in COM motion induced by a perturbation [28]. As such, it remains unclear how arm swing contributes to stability in people with Parkinson’s Disease during slips.

Therefore, this study’s purpose examines differences between the least and most affected legs to slips in people with Parkinson’s Disease with unrestricted and restricted arm swing conditions. We hypothesize that the least affected leg, compared to the most affected, will display a “cautious gait” strategy and increase the distance of the COM’s dynamical state to the edges of the BOS in the mediolateral and anteroposterior directions. We further predict that trunk angular velocities will be larger during the unrestricted arm swing condition (the regular arm motion in people with Parkinson’s Disease) compared to the restricted condition. Finally, we hypothesize an interaction where the distance between the COM’s dynamical state to the BOS will be larger in the least affected leg during the unrestricted arm swing condition compared to the most affected leg when paired with restricted arm swing.

## 2 Methods

### 2.1 Participants

A convenience sample of twenty people with Parkinson’s Disease (13 males and 7 females), ages 48–79 years (63.78 ± 8.97 years) were recruited from the Ottawa-Gatineau community. Since two individuals had severe dyskinesia and one individual had missing data, only 17 participants were included in the final analysis. Participants were assessed with the original Unified Parkinson’s Disease Rating Scale Motor Examination (11± 6) and were between I-III on the Hoehn & Yahr scale [29]. Further, seven participants reported freezing of gait based on the original Freezing of Gait Questionnaire. Participants were tested on their optimally medicated state. Exclusion criteria included any physical discomfort using a virtual reality system, any injuries and/or orthopedic surgeries that interfered with gait, walking only with a walking aid, and additional illnesses other than Parkinson’s Disease. All participants provided written informed consent and the study was approved by the Ottawa Health Science Network Research Ethics Board and the University of Ottawa Research Ethics Board. The study was conducted in accordance with the Tri-Council Policy statement; Ethical Conduct for Research Involving Humans; The International Conference on Harmonization- Good Clinical practice: Consolidated Guideline; and the provisions of the Personal Health Information Protection Act 2004.

### 2.2 Procedure

**Fig 1:**
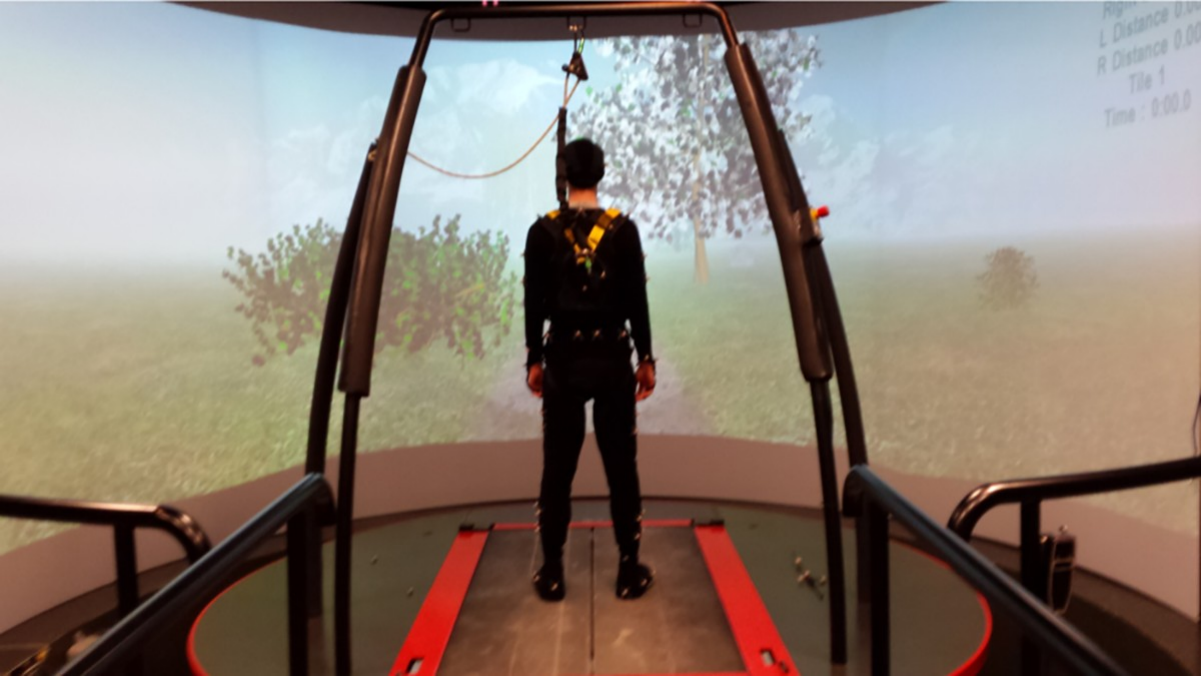
Experimental setup for the CAREN system virtual environment.

A familiarization period was provided for participants prior to data collection to determine their preferred walking speed on the treadmill. Experimental setup is depicted in **Fig 1**. Participants walked at their preferred speed on a dual-belt treadmill with two arm swing conditions (restricted and unrestricted). During the restricted arm swing trials, participants inserted their arms inside the safety harness, which effectively prevented arm motion. The unrestricted arm swing trials were participants’ normal arm swing movements. Arm swing conditions were paired with a slip that was induced on either the least or the most affected side (one slip per trial). This resulted in a total of four trials per participant with each trial lasting 2 minutes. Slips were caused by accelerating the left/right treadmill belt at 1.7m/s^2^ for 0.75 seconds before decelerating at the same rate for 0.75 seconds to return to the participants preferred walking speeds [30]. Slips were automatically triggered, at heel-strike, after the participants’ heels crossed each other during the swing phase in the anteroposterior direction [30]. Twenty-five seconds were provided at the start of each trial for participants to reach steady-state walking before data collection began. To minimize slip anticipation, trials were randomized using a random number generator in Excel 2016 (Microsoft, Seattle, WA, USA) and slip onset within each trial was manually determined in a pseudorandom manner by the CAREN system operator. To ensure safety, and prevent falls, participants wore a safety harness attached to an overhead structure at all times. Participants were encouraged to rest whenever necessary to minimize fatigue.

Data collection was completed using the CAREN-Extended System (Motek Medical, Amsterdam NL) in a virtual park terrain environment. This system combines a six degrees of freedom motion platform with embedded dual-belt instrumented treadmill, 12 camera Vicon Motion Capture system, 180-degree projector screen, and a safety harness. A 57-marker set was used for tracking full body kinematics [15, 31, 32]. Kinematic data were collected at 100Hz.

### 2.3 Data analysis

Markers were synchronized and processed in Vicon Nexus (nexus 2.6, Oxford, UK), while 3D kinematics were calculated in OpenSim with a full-body model [33]. A fourth-order, low-pass Butterworth filter with a 12Hz cutoff frequency was used to filter marker data. Data were analyzed by custom Matlab scripts (MathWorks, Natick, MA) to calculate step time, length, and width, as well as instantaneous linear and angular velocities at heel-strike for both feet. Linear velocities were calculated as absolute values. Additionally, the Margin of Stability (MOS) was calculated bilaterally as a measure of dynamic stability in the anteroposterior and mediolateral directions [34–36]. In the anteroposterior direction, this was calculated as the distance of the extrapolated COM to the right/left heel maker and in the mediolateral direction to the right/left lateral heel marker at heel-strike [15, 31, 36]. Data were categorized by least and most affected legs which were then divided into slipped and recovery limbs. The slipped limb was defined as the leg which was perturbed upon heel-strike, while the recovery limb was defined as the contralateral limb that performed the first heel-strike following the slip.

### 2.4 Statistical analysis

Data were analyzed using SPSS 23.0 (SPSS, IBM, Chicago IL), and p < 0.05 was considered statistically significant. An alpha = 0.05 was set a prioi for statistical significance. The normality of variables was verified using Shapiro-Wilk’s test. A two-way repeated measures ANOVA for arm (unrestricted and restricted) and leg (least and most affected leg) was performed to find main effects and interactions for the slipped limb. Additionally, an identical but separate two-way repeated measures ANOVA assessed main effects and interactions for the recovery limb. If statistical significance was achieved, then pairwise comparisons with a Sidak-Bonferroni adjustment for multiple comparisons were used for post-hoc analyses.

## 3 Results

Slipped limb data for the MOS and spatiotemporal variables are in **Table 1** while linear and angular velocities are in **Table 2**. Recovery limb data for the MOS and spatiotemporal data are in **Table 3** while velocity data are in **Table 4**. Arm range of motion for both arm swing conditions are presented in **Table 5**. Average walking speeds for conditions are included in the **S1 Material**. No differences existed between the legs (p > 0.05) for walking speed. Thus, both sides were averaged before assessing walking speeds between both arm swing conditions with a paired t-test. Results from the t-test revealed no differences in walking speed between the unrestricted and restricted arm swing conditions (p > 0.05).

**Table 1:** Slipped leg average step time, length and width with anteroposterior and mediolateral margin of stability during unrestricted (unrest.) and restricted (rest.) arm swing and for the least and most affected legs.

				P-value	
	Arm	Least Affected	Most Affected	Leg	Arm
AP Margin of Stability(cm)	Unrest.	-20.31±7.12	-20.23±4.66	.503	.336
	Rest.	-18.57±7.00	-20.26±7.06		
ML Margin of Stability(cm)	Unrest.	11.57±3.05	11.00±2.27	.252	.372
	Rest.	11.73±1.69	11.54±2.39		
Step Time(ms)*	Unrest.	436±87	435±44	.691	.006
	Rest.	412±66	426±51		
Step Length(cm)	Unrest.	50.51±11.24	50.58±8.34	.547	.755
	Rest.	49.21±10.53	51.10±8.83		
Step Width(cm)^†^	Unrest.	26.79±4.73	25.75±5.04	.005	.391
	Rest.	27.65±4.30	26.00±4.46		

† Leg Main Effects at p<0.05.

* Arm Main Effects at p<0.05.

**Table 2:** Slipped Leg trunk instantaneous linear and angular velocities in all 3 axes for arm (unrestricted and restricted) and leg (least affected and most affected).

		Unrestricted Arm		Restricted Arm		
		Least Affected	Most Affected	Least Affected	Most Affected	P-Value
Linear Velocity (cm/s) (x10^-2^)	AP	16±6	16±6	18±5	16±6	.15
	ML	4±4	4±3	6±5	4±4	.13
	Vert	18±7	16±4	15±7	16±5	.59
Angular Velocity (°/s) (x10^-2^)	AP	-11±10	-9±8	-17±21	-12±9	.30
	ML	-1±17	-1±10	-1±14	-1±11	.84
	Vert	3±19	-1±10	3±18	0±19	.61

† Leg Main Effects at p<0.05.

* Arm Main Effects at p<0.05.

Values were rounded to the nearest whole number. P-values from the two-way repeated measures ANOVA are reported for leg main effects.

**Table 3:** Recovery leg average step time, length and width with anteroposterior and mediolateral margin of stability during unrestricted (unrest.) and restricted (rest.) arm swing and for the least and most affected legs.

				P-value	
	Arm	Least Affected	Most Affected	Leg	Arm
AP Margin of Stability(cm)^†^	Unrest.	-12.82±0.71	-10.46±0.92	.007	.272
	Rest.	-14.15±0.75	-11.36±0.75		
ML Margin of Stability(cm)	Unrest.	10.46±2.61	10.58±3.08	.416	.063
	Rest.	11.18±2.31	11.80±3.16		
Step Time(ms)^†^	Unrest.	425±111	399±109	.019	.492
	Rest.	437±115	372±97		
Step Length(cm)	Unrest.	56.43±8.90	50.94±11.39	.064	.898
	Rest.	55.56±11.29	52.22±9.98		
Step Width(cm)	Unrest.	22.93±3.84	23.81±5.45	.266	.190
	Rest.	23.92±3.51	24.91±5.49		

† Leg Main Effects at p<0.05.

* Arm Main Effects at p<0.05.

**Table 4:** Recovery Leg trunk instantaneous linear and angular velocities in all 3 axes for arm (restricted and unrestricted) and leg (least affected and most affected).

		Unrestricted Arm		Restricted Arm		
		Least Affected	Most Affected	Least Affected	Most Affected	P-Value
Linear Velocity (cm/s) (x10^-2^)	AP	16±7	17±9	16±6	17±4	.55
	ML	12±8	8±7	9±7	10±8	.29
	Vert	29±10	26±15	26±12	24±19	.52
Angular Velocity (°/s) (x10^-2^)	AP	-30±24	-25±26	-30±25	-29±23	.30
	ML	-12±23	-6±22	-1±28	-3±28	.84
	Vert	-11±50	4±60	-1±50	-2±61	.61

† Leg Main Effects at p<0.05.

* Arm Main Effects at p<0.05.

Values were rounded to the nearest whole number. P-values from the two-way repeated measures ANOVA are reported for leg main effects.

**Table 5:** Average range of motion (degrees) for unrestricted and restricted arm swing conditions for shoulder joint angle in the sagittal plane.

	Unrestricted	Restricted	P-Value
Range of Motion°*	22.63±11.78	4.99±2.26	< .001

* Arm Main Effects at p<0.05.

### 3.1 Arm swing

As no statistical significance (p > 0.05) in range of motion (ROM) existed between the least and most affected arms, the two arms were averaged for further analysis. The paired samples t-test revealed that the restricted arm swing condition had a reduced arm ROM than the unrestricted arm swing condition (t(16) = -6.13, p < 0.001). The ANOVA revealed that participants had a faster step time (F(1,16), p = 0.006, η_p_^2^ = 0.389) during the restricted arm swing condition compared to unrestricted arm swing during heel-strike of the slipped limb. No further arm swing main effects or interactions were found.

### 3.2 Least and most affected legs

Our results demonstrated that when the least affected leg was the slipped limb, it had a wider step than the most affected (F(1,16) = 10.788, p = 0.005, η_p_^2^ = 0.403). Further, when the least affected leg was the recovery limb, it had a larger anteroposterior MOS (F(1,16) = 9.654, p = 0.007, η_p_^2^ = 0.376), a longer step time (F(1,16) = 6.825, p = 0.019, η_p_^2^ = 0.299), and had a trend for a longer step length (F(1,16) = 3.955, p = 0.064, η_p_^2^ = 0.198) than the most affected. No additional main effects or interactions were found.

## 4. Discussion

This study examined the effect of unrestricted and restricted arm swing conditions during treadmill slips on the least and most affected legs in people with Parkinson’s Disease. Our results did not support our hypothesis that the unrestricted arm swing condition would improve dynamic stability compared to the restricted arm swing condition. Rather, no differences existed between our arm swing conditions for the MOS nor for velocities. This suggests that, even when available, participants did not effectively engage an arm elevation strategy to recover dynamic stability after the slip. However, our participants had a faster step time on the leg undergoing the slip during the restricted arm swing condition. Aligned with our hypothesis, foot placement and slip recovery responses were asymmetric in our participants. Indeed, our participants had a larger step width on their least affected leg compared to their most affected for the slipped limb. Further, during the recovery step, our participants reduced the risk of backward stability loss on their least affected leg by increasing this leg’s anteroposterior MOS and step time.

### 4.1 Arm swing

Contrary to our hypothesis, there were no differences between the unrestricted and restricted arm swing conditions neither for the MOS nor linear and angular velocities. This occurred despite differences in arm ROM between our arm swing conditions. These findings were unexpected due to the functional role arm swing has in the recovery strategy to slips [16–18]. Marigold et al. [16, 17] demonstrated that in response to a slip, healthy individuals execute a rapid arm elevation response where the arms are flexed forward and upward. The authors discussed that the arms flex specifically in these directions to move the COM further anteriorly and in the opposite direction of the trunk’s initial backward displacement caused by the slip [16]. An anterior shift in the COM’s position would theoretically be reflected as an increase in the anteroposterior MOS as it measures the distance of the COM’s dynamical state to the BOS [10, 36]. This in turn would reflect that the arm elevation strategy is effective in reducing dynamic stability loss in the backward direction [16–18]. However, when our MOS results are examined alongside our linear and angular velocities, the lack of findings indicate that our participants’ arm movements did not influence their trunk kinematics. One potential explanation for this is that our participants may have been ineffective in executing a targeted arm elevation response to the slip. Carpenter et al. [6] demonstrated that the arm responses in people with Parkinson’s Disease during platform perturbations were more variable in their trajectory and had a shorter response time compared to controls. Further, the arms returned to a position near their trunk relatively quickly in people with Parkinson’s Disease [6]. The authors further found that the recovery arm responses in people with Parkinson’s Disease were particularly ineffective and abnormal when perturbations were induced in the backward direction (the same destabilizing direction for slips) [6]. This could explain the lack of findings in our study between our arm swing conditions for the slipped and recovery limb analyses. However, further research is needed to discriminate the impact of age and Parkinson’s disease on arm strategies in response to slips. Carpenter et al. [6] found that the arm response to perturbations was ineffective despite the deltoid muscles activating earlier in people with Parkinson’s Disease than controls. Therefore, future research should examine the EMG profile of the deltoid muscles between these two groups.

Interestingly, the leg undergoing the slip had a faster step during the restricted arm swing condition compared to the unrestricted. This response could reflect destabilization from the restricted arm swing condition in our participants as it may have impeded their movement timing [15]. Indeed, a faster step reduces the amount of time the contralateral leg (recovery limb) has to determine appropriate foot placement. Previous research suggests that walking with restricted arm swing disrupts the rhythmic temporal sequence of foot placement in this demographic [15]. One of the primary symptoms of Parkinson’s Disease is impaired internal movement timing [37]. Therefore, our finding supports the notion that when arm swing becomes completely absent (due to the disease), foot placement timing is further disrupted [15]. As such, disruption to temporal foot placement arises both mechanically from the absent arm swing and neurologically from the Basal Ganglia’s neurodegeneration [15].

### 4.2 Least and most affected leg

As hypothesized, our analysis revealed differences between the least and most affected leg. This potentially reflects the asymmetric neurodegeneration in people with Parkinson’s Disease. Indeed, our participants demonstrated asymmetric impairment to mediolateral foot placement as they had a wider step on their least affected leg, compared to the most affected, when the least affected leg was undergoing the slip. When stability is threatened, both healthy individuals and people with Parkinson’s Disease widen their steps to maintain the COM within the lateral boundaries of the base of support [15, 38]. However, during walking, the neuromuscular system determines foot placement by predicting the COM’s future position at the upcoming heel-strike [39–41]. In our study, slip occurrence was randomized between the legs and slip onset within each trial, was established in a pseudorandom manner. This minimized the possibility for our participants to predict the timing and laterality of the perturbation. Thus, the least affected leg’s larger step width (at slip onset) reflects an already existing step asymmetry prior to the perturbation. Since our study did not examine differences between the first and subsequent slips, step width differences potentially arose as participants attempted to widen their BOS after initial slip exposure. However, the results indicate that our participants were more effective in adjusting the BOS mediolaterally with their least affected leg.

In addition, asymmetric neurodegeneration would account for the differences between the legs in attenuating backward stability loss. Indeed, the longer step time and larger anteroposterior MOS when the least affected leg was the recovery limb would reflect appropriate and asymmetric slip responses. A longer step time provides more time for stable foot placement of the contralateral leg before the COM is transferred between limbs. Additionally, the larger anteroposterior MOS reduces the risk of backward stability loss by moving the COM’s dynamical state further anteriorly and in the opposite direction to the slip [10, 20, 21]. As step time and anteroposterior COM movement are regulated passively by subcortical structures, the differences between the legs may reflect the asymmetric neurodegeneration of dopamine within the Basal Ganglia [10, 24, 25]. These interlimb differences hold important implications for clinicians. Specifically, clinicians should consider the efficacy of targeting the least affected leg to promote gait adaptation and stability in slip recovery responses. This would be in line with initial evidence from Ricciardi et al. [42], who demonstrated in a pilot study that physical therapy targeting the least affected side improved more in the UPDRS-III and Tinetti scale subscores than standard therapies targeting both legs.

### 4.3 Limitations

Several limitations should be considered in the context of our study. For instance, participants were tested on their optimally medicated state which affects foot placement [37]. Additionally, differences between freezers and non-freezers were not examined. As freezers demonstrate greater postural instability than non-freezers, restricted arm swing may have a distinct effect on their dynamic stability [43, 44]. Future research should also consider examining differences between people with Parkinson’s Disease and healthy aged-matched controls to parse out differences due to age and those that arise due to Parkinson’s Disease. In addition, it has been shown that after perturbation exposure, healthy adults adopt a more cautious gait i.e. with shorter, wider, and slower steps as a means to enhance their dynamic stability to prevent potential future perturbations [10, 22]. It is possible that our participants used a similar strategy when walking after initial slip exposure. Therefore, this should be taken into account when interpreting the findings of this article. Similarly, individuals with a higher Hoehn & Yahr score may respond differently to slips than those with a lower score as these individuals have further reductions in their postural control. Research on perturbation recovery responses in people with Parkinson’s Disease would also benefit from an examination of the harness load data, an aspect unexamined in our study, to assess balance loss versus recovery. Further, future studies should examine differences in the recovery response between the first slip and following slips since differences may occur in the proactive and reactive recovery responses in restoring dynamic stability. Finally, this study did not examine differences between the dominant and non-dominant leg which may elicit further differences in response to slip recovery.

## 5. Conclusion

No differences between our arm swing conditions suggests that the normal arm swing in our participants was not more effective at restoring stability after an induced slip compared to when their arm motion was restricted. Lack of differences plausibly arose from our participants ineffectively implementing the arm elevation strategy to move their COM in the opposite direction from the slip’s backward displacement despite differences in arm ROM between the two arm swing conditions. To determine if the arms follow an uncoordinated trajectory to counteract slip destabilization in people with Parkinson’s Disease, future research should compare differences between these individuals and healthy elderly adults. Contrastingly, to our arm swing results, the increased anteroposterior MOS, increased step time and width in the least affected leg, compared to the most, suggest that our participants were asymmetrically impaired in executing their slip recovery responses. Differences between the legs may reflect the asymmetric neurodegeneration that occurs in this demographic. Since mobility is more intact in the least affected leg, our participants adjusted foot placement of this limb to mitigate the destabilizing effects of the slip to recover stability. As such, clinicians should consider therapies that facilitate adaptive responses in the least affected leg as mobility may be too impaired in the most affected leg for effective perturbation recovery.

## Supporting information

S1 Material Average walking speed for Unrestricted and Restricted arm swing conditions. (DOCX)

S1 Data (XLSX)
