# Supplementary material for: Recovery of dynamic stability during slips unaffected by arm swing in people with Parkinson’s Disease
Source: PLoS One. 2021 Apr 6;16(4):e0249303. doi: 10.1371/journal.pone.0249303 (PMC8023478; doi:10.1371/journal.pone.0249303)
Supplement: S1 Material — (DOCX) [file pone.0249303.s001.docx]

**S1 Material**. Average walking speed for Unrestricted and Restricted arm swing conditions.

|  | Unrestricted | Restricted | P-Value |
| --- | --- | --- | --- |
| Walking Speed (m/s) | 0.99±0.16 | 1.00±0.15 | 0.771 |

* Arm Main Effects at p<0.05
